# SNP Genotype Imputation in Forensics—A Performance Study

**DOI:** 10.3390/genes15111386

**Published:** 2024-10-28

**Authors:** Andreas Tillmar, Daniel Kling

**Affiliations:** 1 Department of Forensic Genetics and Forensic Toxicology, National Board of Forensic Medicine, SE-58758 Linköping, Sweden; daniel.kling@rmv.se; 2 Department of Biomedical and Clinical Sciences, Faculty of Health Sciences, Linköping University, SE-58183 Linköping, Sweden; 3 Department of Forensic Sciences, Oslo University Hospital, NO-0424 Oslo, Norway

**Keywords:** forensic genetics, forensic investigative genetic genealogy, kinship analysis

## Abstract

Background/Objectives: Emerging forensic genetic applications, such as forensic investigative genetic genealogy (FIGG), advanced DNA phenotyping, and distant kinship inference, increasingly require dense SNP genotype datasets. However, forensic-grade DNA often contains missing genotypes due to its quality and quantity limitations, potentially hindering these applications. Genotype imputation, a method that predicts missing genotypes, is widely used in population and medical genetics, but its utility in forensic genetics has not been thoroughly explored. This study aims to assess the performance of genotype imputation in forensic contexts and determine the conditions under which it can be effectively applied. Methods: We employed a simulation-based approach to generate realistic forensic SNP genotype datasets with varying numbers, densities, and qualities of observed genotypes. Genotype imputation was performed using Beagle software, and the performance was evaluated based on the call rate and imputation accuracy across different datasets and imputation settings. Results: The results demonstrate that genotype imputation can significantly increase the number of SNP genotypes. However, imputation accuracy was dependent on factors such as the quality of the original genotype data and the characteristics of the reference population. Higher SNP density and fewer genotype errors generally resulted in improved imputation accuracy. Conclusions: This study highlights the potential of genotype imputation to enhance forensic SNP datasets but underscores the importance of optimizing imputation parameters and understanding the limitations of the original data. These findings will inform the future application of imputation in forensic genetics, supporting its integration into forensic workflows.

## 1. Introduction

Genotype imputation is a statistical method used to predict missing genotypes from observed genotype data [1,2]. This technique relies primarily on the principle of allelic association, commonly referred to as linkage disequilibrium (LD). By utilizing the LD structure from phased haplotype reference panels, which comprise samples with dense genetic marker maps, genotype imputation has become a useful tool in genetic research. It has been extensively studied and applied in various fields such as population genetics, genome-wide association studies (GWASs), medical genetics, and more [3,4,5]. Despite the proven utility of genotype imputation, its potential in forensic applications remains relatively underexplored and has only been investigated in a limited number of studies so far [6,7,8,9].

The core principle of genotype imputation involves the prediction of missing genotypes by using shared DNA segments among individuals. These segments are conserved over generations due to high levels of LD and low recombination rates. In practice, genotype data from both the test samples (i.e., datasets to be imputed) and the reference individuals are first converted into a haploid format (i.e., haplotypes) using phasing methods [10]. Phasing software often integrates both phasing and imputation, employing Hidden Markov Models (HMMs) to probabilistically determine the most likely haplotype configurations. Once phasing is completed, the missing genotypes in the test samples can be predicted by identifying matching haplotypes in the reference panel. The genotype predictions, both phasing and imputation, are not always perfect, and many of the existing imputation models and software provide a prediction probability along with the imputed genotype, which corresponds to the uncertainty of the imputed genotype variants.

The accuracy and performance of genotype imputation are influenced by several key factors, which determine its utility in various applications. Examples of such factors include the following: (1) The quality and density of the observed genotypes [11]. High-quality, densely genotyped data increase imputation accuracies. (2) They also include reference data characteristics [3,12,13]. The size and genetic composition of the reference panel are critical determinants of imputation accuracy. Larger reference panels, which capture a wide range of genetic diversity, provide a more comprehensive basis for predicting missing genotypes. Additionally, reference panels that closely match the genetic background of the target population enhance the accuracy of imputation, as they are more likely to share relevant haplotypes with the test samples. (3) Minor allele frequency [1,3,12,14]. The frequency of alleles plays a significant role in imputation performance. Common alleles, which are more frequently observed in the population, tend to be imputed with higher accuracy due to the abundance of LD information. In contrast, rare alleles, which have lower levels of LD than common variants, are imputed with greater uncertainty, as they are less frequently represented in the reference data. Furthermore, alleles that are not present in the reference data will not be predicted.

There are several potential applications of genotype imputation in forensic genetics. Forensic investigations often involve samples of poor-quality or degraded DNA, where genotype datasets with a large proportion of missing SNP genotypes may be common and hinder downstream analysis. The most apparent use of genotype imputation is in FIGG cases [8] and when trying to identify distant relationships [9]. In these situations, SNP-to-SNP imputation may be applied to both low-quality datasets, but also to higher-quality datasets obtained through targeted approaches that lack the necessary SNPs. In scenarios where low-read-depth sequencing data are obtained, imputation can compensate by increasing the number of SNP markers available, even if the quality of individual SNPs is low. A two-step approach can both help generate more comprehensive genetic profiles from low-coverage sequencing data, but also increase the genotype quality of the observed data that are often encountered in forensic samples [15].

The most commonly used genetic marker in forensic genetics is Short Tandem Repeats (STRs). These are the gold standard when it comes to one-to-one matching to compare evidentiary samples from a crime scene with samples from suspects [16]. Imputing STR profiles from SNP profiles may be a desirable future application. While the exact matching of STR profiles using SNP data may not be possible due to the much higher diversity of STR alleles, probabilistic approaches may be possible in the future. Studies by Edge et al. [17] and Kim et al. [6] have demonstrated that it is plausible to impute genome-wide SNP data from standard STR profiles, although the success and accuracy of such methods are still limited.

Different forensic applications are more or less sensitive to genotype errors, and it is crucial to know the extent of these when using genotype imputation. To address this, it is essential to understand the factors that influence imputation performance and to be able to optimize its use in forensic genetics. The aim of the current study was to conduct a performance analysis of genotype imputation for forensic applications. Specifically, we focused on SNP-to-SNP imputation, aiming to establish extended SNP profiles from partial SNP datasets for FIGG or extended kinship analysis.

## 2. Materials and Methods

### 2.1. Test Samples

Ten CEPH-UTAH (CEU, EUR) and Ten Dai Chinese (CDX, EAS) genome sample sets were randomly selected from the 1000 Genomes Project [18] as test samples. These samples were subsequently removed from the genotype imputation reference dataset. Although larger sample numbers may improve precision regarding rare variants, prior studies have shown that small but diverse sample sets can provide reliable imputation results [19]. For all test samples, complete SNP genotype datasets were extracted based on 1.3 million SNPs. This set of SNPs is relevant for FIGG applications and extended kinship analysis [20]. Pruned SNP datasets corresponding to 4000, 10,000, 50,000, 100,000, and 300,000 SNPs were generated by random sampling from the original SNP datasets for each of the test samples. This number of markers reflects plausible targeted SNP panel sizes used in forensics genetics (e.g., [21,22,23]). All imputation tests were conducted on SNPs located specifically on chromosome 22, rather than across all chromosomes. This approach was chosen for efficiency purposes, as working with a single chromosome allows for more manageable and quicker analyses. The imputation results obtained from the analyses of chromosome 22 were then extrapolated to apply to all chromosomes. This approach of focusing on one chromosome is commonly used for its practicality and effectiveness in handling large-scale genetic data [24,25]. For reference, imputation was performed on all chromosomes for two samples. 

To study the impact of genotype errors in the observed genotype datasets, errors were introduced in the form of allelic drop-in (at a 0.1 drop-in rate) and allelic drop-out (at a 0.1 drop-out rate). Drop-in was limited to homozygous genotypes.

### 2.2. Imputation

Genotype imputation was performed with the software Beagle version 5.2 [2,26]. Although other imputation software is available, Beagle remains competitive for a wide range of datasets. Comparative studies have shown that Beagle performs well across diverse populations and imputation scenarios [27,28]. The software Conform (https://faculty.washington.edu/browning/conform-gt.html, accessed on 1 October 2024) (version “24May16”) was applied prior to the imputation in order to check the file format, consistencies between the target and reference SNP definitions, allele definitions, and other parameters. Genotype data from the 1000 Genomes Project (including all populations, “ALL”, (http://bochet.gcc.biostat.washington.edu/beagle/1000_Genomes_phase3_v5a/, accessed on 1 October 2024) were used as the reference dataset unless otherwise stated. The sample to be imputed was removed from the reference dataset prior to imputation. Beagle was run with the following parameter settings: burn in = 6, iterations = 12, phase-states = 280; imp-states = 1600, imp-segment = 6.0, imp-step = 0.1, imp-n steps = 7, cluster = 0.005, ap = true, gp = true, ne = 1,000,000, window = 400 cM (HapMap GrCh37 map: https://bochet.gcc.biostat.washington.edu/beagle/genetic_maps/, accessed on 1 October 2024), and overlap = 4.0. These values were chosen because they are the optimized defaults recommended by the Beagle developers, balancing imputation accuracy with computational efficiency, and have been used in various studies [19]. We refer to the manual of Beagle for a short explanation of the parameters. Imputed genotypes were added to the set of observed genotypes if the genotype imputation probability met or exceeded the threshold Q_gp_. Different datasets were created with the parameter Q_gp_ set to 0.5, 0.9, 0.95, or 0.99, respectively.

### 2.3. Performance Tests and Statistics

The imputation study was designed to study how various factors affect the imputation performance, such as call rate and accuracy. These included the number, density and quality of the observed genotypes (e.g., genotypes used as the input for the imputation), reference data, reference populations, and genotype errors in the input data.

To evaluate imputation performance, we used two metrics: call rate and error rate. The call rate is the proportion of assigned genotypes, defined as the sum of observed and imputed genotypes divided by the total targeted genotypes. A higher call rate reflects greater coverage of the dataset. The error rate is the proportion of incorrectly assigned genotypes, calculated as the number of erroneous genotypes divided by the total number of observed and imputed genotypes. Both metrics are commonly used in genotype imputation studies to assess imputation quality [19,29]. Higher accuracy in genotype imputation is typically associated with higher call rates and lower error rates.

Call rate and error rate variations were assessed using the medians, minimums, and maximums for the tested samples.

## 3. Results and Discussions

This study aimed to analyze the performance of basic SNP-to-SNP genotype imputation in forensic applications, with a focus on extending SNP profiles from partial datasets for FIGG or extended kinship analysis. 

**Figure 1:**
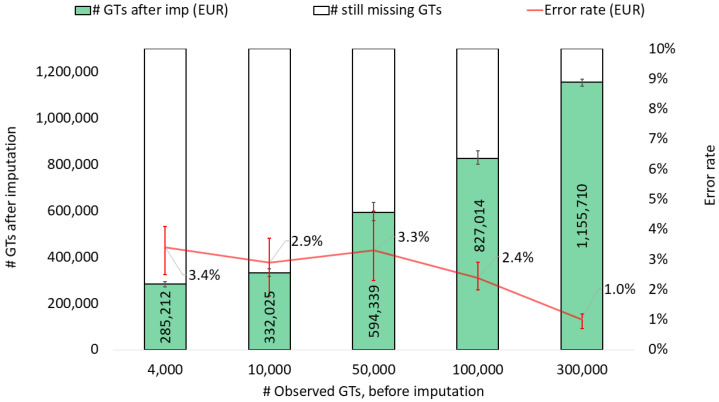
Imputation performance (number of SNP genotypes (GTs) after imputation and genotype error rate) when imputing from a variable number of observed genotypes. The data are based on the imputation of 10 EUR samples, with values representing medians and bars representing minimum and maximum values. A calling threshold of genotype imputation probability, per SNP, of >0.95 was applied.

First, we studied the general impact of the number of observed SNP genotypes on genotype imputation. As the number of observed genotypes increased, the number of imputed genotypes also increased, while the total error rate concurrently decreased (Figure 1). The results indicated overall that genotype imputation performed reasonably well, even when starting with as few as 4000 observed genotypes. In this scenario, it was possible to impute up to 300,000 genotypes by applying a calling threshold of >0.95 for the genotype imputation probability. However, the error rate was relatively high, with a median of 3.4%. Starting with 100,000 observed genotypes resulted in an approximately 8-fold increase in the number of imputed genotypes, with a median error rate of 2.4%. Such a number of genotypes could well be sufficient for both FIGG and extended kinship analysis [30]. The overall trend of the data underscores that increasing the number of initial SNP observations improves the call rate and accuracy of the imputation process. This finding is particularly important in forensic contexts, where both the accuracy and completeness of genotype data can be critical.

When comparing these results with other imputation studies, our error rates appear lower [12,15,31]. There are several plausible explanations for this. One reason could be that our SNP targets contain relatively few rare alleles (medians around 0.1%, with the vast majority above 0.01%; see Figure S1). As highlighted in the previous literature, rare alleles are much harder to impute accurately, often necessitating larger reference panels to improve precision [14]. In forensic applications, this difference may be significant, as rare alleles could play a crucial role and the lower presence of rare alleles in our dataset may have contributed to the higher imputation accuracy observed. 

**Figure 2:**
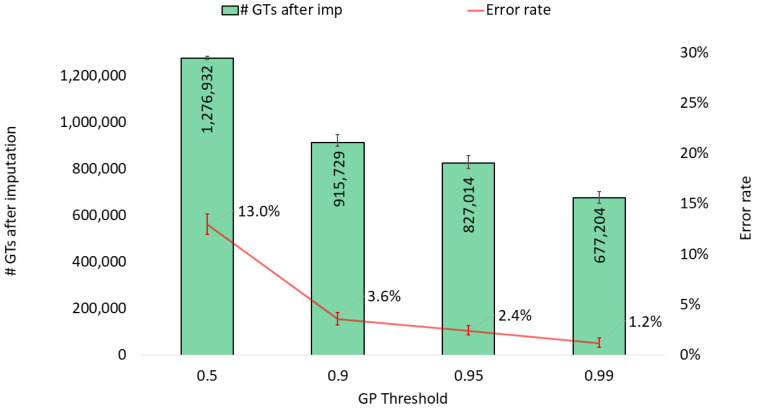
Imputation performance (number of SNP genotypes (GTs) after imputation and genotype error rate) when applying different calling thresholds for genotype imputation probability (GP threshold). The data are based on the imputation of 10 EUR samples using 100,000 observed genotypes, with values representing medians and bars representing minimum and maximum values.

Another reason for the observed improvement in the accuracy of our targeted SNP panel may be the application of a calling threshold for genotype imputation probability, which increases the certainty of the called genotypes. The choice of threshold may have implications for further analysis and is of interest to examine. The genotype imputation probability is provided for each imputed SNP genotype and indicates how well the imputed genotype fits the reference data and the applied model parameters. Thus, the imputation probability can be interpreted as the uncertainty of the imputed genotype. This probability, ranging from 0 to 1, can be used as a calling threshold to balance call rate and accuracy when performing imputation. The choice of threshold has previously been shown to have a significant impact on the performance [24]. Figure 2 shows how the call rate and the error rate decreased in our test data when increasing the threshold. It is clear that the choice of threshold also has a considerable impact on our targeted SNPs, both in terms of call rate and error rate. For example, the error rate has a median of 13% when using an arbitrary threshold (0.5), while it drops to 1% with a threshold of 0.99. For certain forensic applications, it may be crucial to keep the error rate as low as possible, whereas for other applications, it is more important to obtain the highest possible SNP coverage without deference to high error rates [32].

**Figure 3:**
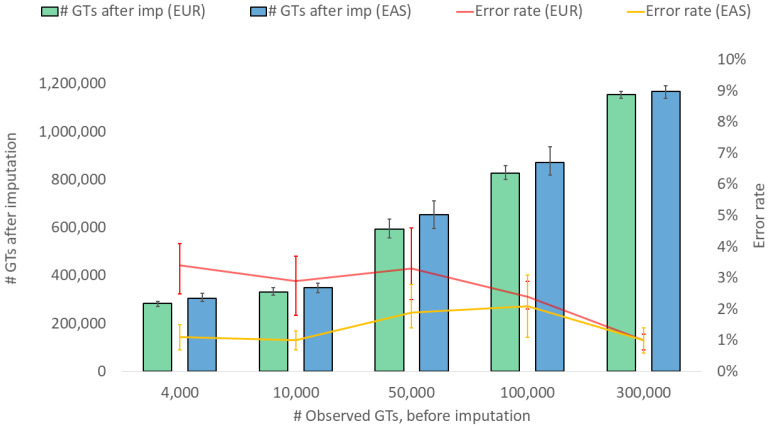
Imputation performance (number of SNP genotypes (GTs) after imputation and genotype error rate) for European (EUR) and East Asian samples (EASs) when imputing from a variable number of observed genotypes. The values represent medians, and the bars represent minimum and maximum values. A calling threshold of genotype imputation probability, per SNP, of >0.95 was applied.

Earlier studies have shown that imputation performance may vary between populations due to differences in LD patterns and different haplotype frequencies [13,31,33]. These population-specific variations are particularly relevant in forensic genetic contexts, where the biogeographic origin of the individual from whom the sample originates is often unknown. This uncertainty highlights the importance of examining potential differences in imputation accuracy across populations, as such differences may affect the reliability of forensic analyses, including ancestry inference, kinship analysis, and identification. To address this, we compared the genotype imputation performance between samples of European ancestry to those of East Asian ancestry separately. Figure 3 shows that a similar number of imputed genotypes was obtained for both European and East Asian samples with a 0.95 genotype probability threshold. The error rates were, on average, lower for the East Asian samples compared to the European samples at lower levels of observed genotypes (50,000 or less). 

Interestingly, Huang et al. [31] found a slightly higher accuracy, i.e., lower error rates, for EASs than for EUR in their study, while Das and colleagues found overall less accuracy for EASs compared with EUR [3]. This shows that the circumstances (SNP targets, reference panel, etc.) of the imputation play a crucial role and that the context is important to consider.

**Figure 4:**
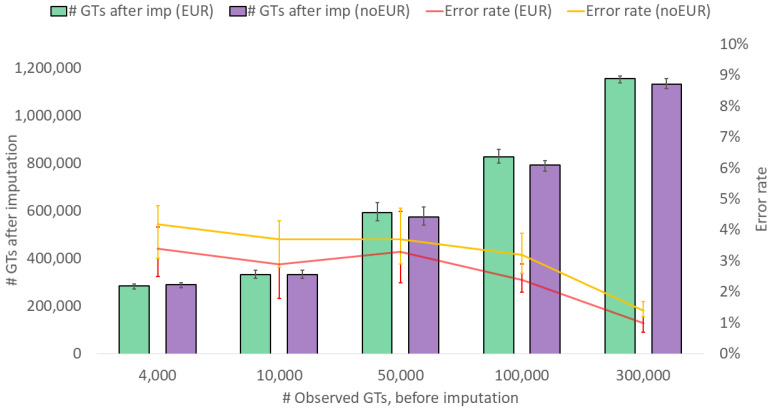
Imputation performance (number of SNP genotypes (GTs) after imputation and genotype error rate) for European (EUR) samples when reference samples from other European samples are included (EUR) and excluded (noEUR), imputing from a variable number of observed genotypes. The values represent medians, and the bars represent minimum and maximum values. A calling threshold of genotype imputation probability, per SNP, of >0.95 was applied.

In the context of potential population-specific factors, it is also important to understand how the performance may be affected when relevant population samples are missing from the reference dataset. Figure 4 shows that the number of genotypes after imputation is similar, whether or not reference individuals from the same population as the samples to be imputed are included. However, the error rates are, on average, slightly higher when reference samples from the same population are missing. This result is not unexpected, and the same tendency has been shown before [34]. Huang and colleagues [31] performed imputation from samples originating from small rural populations, which showed that the accuracy differed substantially between the tested populations. This highlights the importance of considering the composition of the reference panel in relation to the population ancestry of the samples being imputed. In forensic genetics, samples of unknown origin are often handled, and it may therefore be important to perform a dedicated analysis of biogeographical ancestry prior to imputation to determine the composition of the reference dataset [35]. 

**Figure 5:**
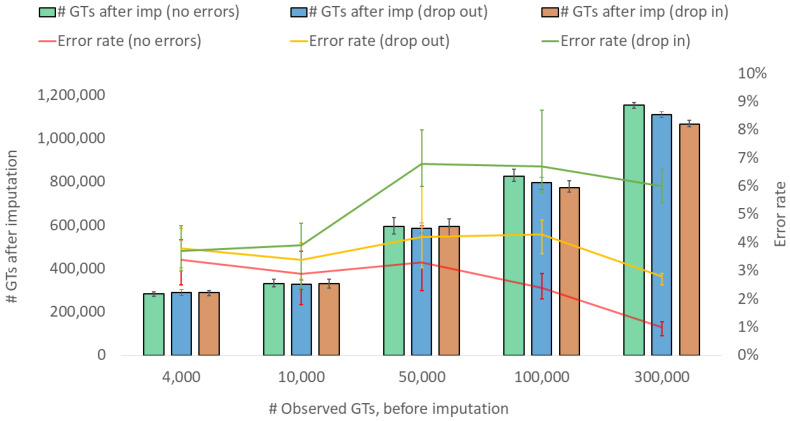
Imputation performance (number of SNP genotypes (GTs) after imputation and genotype error rate) when imputing from a variable number of observed genotypes and different error types (allelic drop in, allelic drop out, and no errors) in the observed datasets. The data are based on the imputation of EUR samples, with values representing medians and bars representing minimum and maximum values. A calling threshold of genotype imputation probability, per SNP, of >0.95 was applied.

The results presented thus far are based on analyses of observed genotype data without genotype errors. In many forensic genetic applications, the sample quality may be poor, increasing the risk of errors among the observed genotypes [36,37], especially for low-coverage sequencing [22]. When imputing from samples containing genotype errors, the call rates decreased slightly for the higher levels of observed genotypes (>50,000 observed genotypes), for both drop-in and drop-out error types (Figure 5), compared with imputing from samples with no genotype errors. The imputation error rates, measured after imputation, were on average higher for both drop-in and drop-out error types, being more significant when there were drop-in genotype errors in the observed genotype dataset. Observed drop-in errors result in false heterozygote genotypes, increasing the risk of imputing false heterozygotes located adjacent to the observed genotype, whereas, in contrast, alleles dropping out will potentially have less effect on the accuracy. Differentiating errors and their impact on imputation accuracy have not been widely studied and should be addressed in future studies. As demonstrated in a previous study, the genotype likelihood input mode in Beagle can be used along with filtering for confident genotypes to impute the missing genotypes (e.g., a two-step approach). This procedure, when tested on ancient genomes, outperformed a single-step imputation from genotype likelihoods [15].

This imputation study was conducted using input genotypes and targets located on chromosome 22, after which the result was interpolated to represent performance across all chromosomes. To assess the validity of this approach, genotype imputation was performed on two samples with data and targets across all 22 chromosomes. When comparing the results, a small difference was detected (a mean difference of approximately 10%), which was not unexpected and demonstrated that genotype imputation performance may vary between chromosomes.

**Figure 6:**
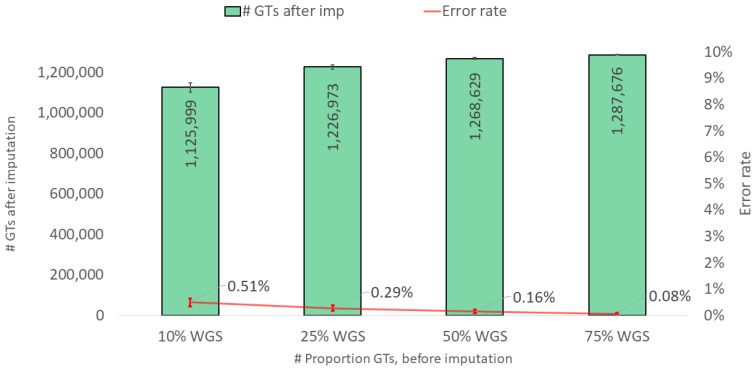
Imputation performance (number of SNP genotypes (GTs) after imputation and genotype error rate) when imputing from a variable proportion of observed genotypes simulating WGS datasets. The results are based on the imputation of 10 EUR samples, with values representing medians and bars representing minimum and maximum values. A calling threshold of genotype imputation probability, per SNP, of >0.95 was applied.

The current study primarily focused on analyzing observed and missing genotype data for a targeted SNP panel (e.g., microarray SNP chip, capture hybridization sequencing), employing commonly used SNP typing methods. An alternative approach is to perform whole-genome sequencing (WGS) and then extract the SNP genotypes of interest from virtual SNP panels. With WGS data, a greater number of observed SNP genotypes would likely be available, which could, in turn, improve the performance when imputing the missing genotypes. Although this was outside the main scope of this study, we conducted an analysis emulating WGS datasets where 10% to 75% of genome-wide SNPs were available as input. As expected, this approach generated significantly higher call rates compared to the targeted approach studied earlier (Figure 6), with error rates decreasing tenfold. This aligns with the findings of Li et al. [38], who demonstrated that WGS-derived data provide enhanced coverage and accuracy for imputation, particularly for rare variants that are often missed by targeted SNP arrays. While WGS may still be more expensive than targeted approaches, it may be worth considering if higher accuracy is required; however, this holds true only as long as the WGS data do not contain significant levels of errors. The forensic applications of WGS are worth considering, especially in scenarios requiring high levels of accuracy. For example, the accurate imputation of missing genotypes in degraded or low-quality DNA samples is often crucial in forensic contexts, and the higher call rates observed with WGS may make this approach particularly valuable. The comprehensive genomic coverage offered by WGS could help mitigate the risks of false positives or erroneous exclusions, which can arise from incomplete or biased SNP panels [39].

## 4. Conclusions

Genotype imputation is a valuable statistical method that predicts missing genetic variants in a dataset. This technique has been widely applied in fields such as medical genetics, population genetics, and genome-wide association studies. In forensic genetics, where missing data are common due to the low quantity and quality of DNA samples, SNP genotype imputation could serve as a powerful method. Our study demonstrates that imputation can effectively extend genotype datasets with substantial missing data. However, imputation is not without limitations. Errors, particularly in rare variants or poorly covered regions, can be introduced, which may impact downstream applications. It is therefore essential to carefully consider the potential impact of these errors, especially in sensitive applications such as ancestry and phenotype predictions, whereas errors may be less critical in extended kinship analysis.

## Data Availability

Data are stored at the NBFM and may be made available to approved laboratories upon written request.

## Supplementary Materials

The following supporting information can be downloaded at https://www.mdpi.com/article/10.3390/genes15111386/s1: Figure S1: MAF distributions for SNP targets.
