# Supplementary material for: SNP Genotype Imputation in Forensics—A Performance Study
Source: Genes (Basel). 2024 Oct 28;15(11):1386. doi: 10.3390/genes15111386 (PMC11593911; doi:10.3390/genes15111386)
Supplement: Supplementary file 1 [file genes-15-01386-s001.zip › Supplementary Figure S1.pdf]

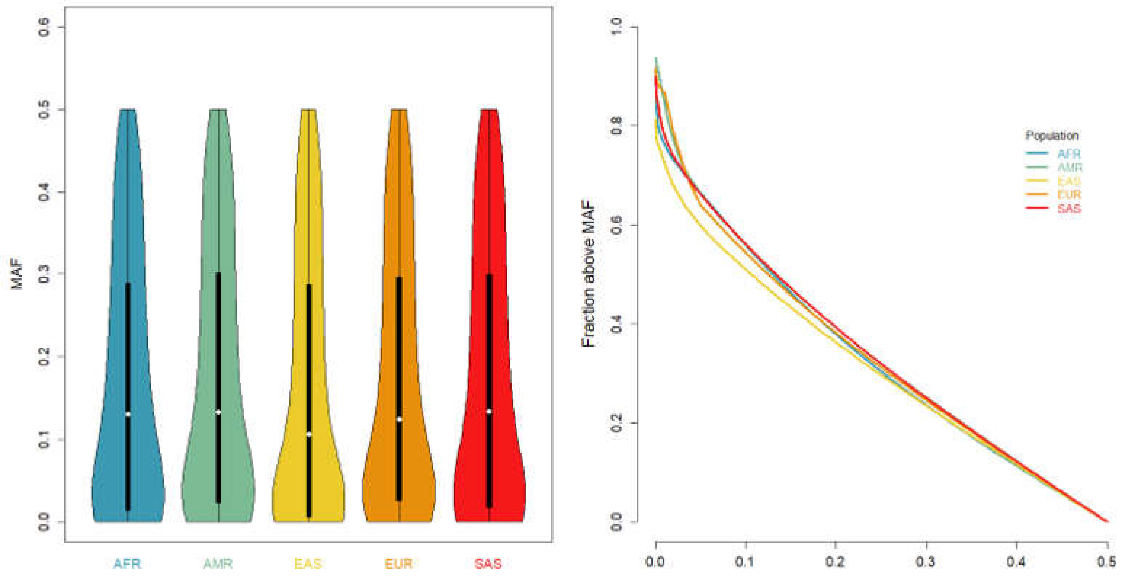

Figure S1. MAF distributions for SNP targets**.** MAF distribution for the 1000 Genomes super populations for the 1.3M SNP targets included in the study.
